# Crosstalk between breast cancer-derived microRNAs and brain microenvironmental cells in breast cancer brain metastasis

**DOI:** 10.3389/fonc.2024.1436942

**Published:** 2024-08-08

**Authors:** Munazza S. Khan, Grace L. Wong, Chuling Zhuang, Mariana K. Najjar, Hui-Wen Lo

**Affiliations:** ^1^ Vivian L. Smith Department of Neurosurgery, McGovern Medical School, The University of Texas Health Science Center at Houston, Houston, TX, United States; ^2^ The University of Texas MD Anderson Cancer Center UTHealth Houston Graduate School of Biomedical Sciences, The University of Texas Health Science Center at Houston, Houston, TX, United States; ^3^ Department of Integrative Biology and Pharmacology, McGovern Medical School, The University of Texas Health Science Center at Houston, Houston, TX, United States

**Keywords:** breast cancer, microRNA, brain microenvironment, blood-brain barrier, brain metastasis

## Abstract

Breast cancer is the most frequent malignancy in women, constituting 15.2% of all new cancers diagnosed in the United States. Distant breast cancer metastasis accounts for the majority of breast cancer-related deaths; brain metastasis is the third most common site for metastatic breast cancer but is associated with worst prognosis of approximately eight months of survival. Current treatment options for breast cancer brain metastasis (BCBM) are limited and ineffective. To help identify new and effective therapies for BCBM, it is important to investigate the mechanisms by which breast cancer cells metastasize to the brain and thrive in the brain microenvironment. To this end, studies have reported that primary breast tumor cells can prime brain microenvironmental cells, including, astrocytes and microglia, to promote the formation of BCBM through the release of extracellular vesicle-microRNAs (miRNAs). Breast tumor-derived miRNAs can also promote breast cancer cell invasion through the blood-brain barrier by disrupting the integrity of the brain microvascular endothelial cells. In this review, we summarize current literature on breast cancer-derived BCBM-promoting miRNAs, cover their roles in the complex steps of BCBM particularly their interactions with microenvironmental cells within the brain metastatic niche, and finally discuss their therapeutic applications in the management of BCBM.

## 1 Introduction

According to the latest statistics by the American Cancer Society, the estimated number of new breast cancer cases in 2024 is 310,720, accounting for 32% of all cancers diagnosed in women (1). Breast cancer patients have the second highest rate of brain metastasis behind lung cancer (2). Furthermore, subtype analysis of breast cancer brain metastasis (BCBM) patients showed that HER2-enriched breast cancer and triple-negative breast cancer (TNBC) subtypes have a higher potential to develop brain metastasis, underscoring the particular importance of studying BCBM in these two subtypes of breast cancer (3).

In 1993, Ambros and his team made the groundbreaking discovery of a microRNA (miRNA) in *Caenorhabditis elegans* (4). The miRNA, transcribed from the lin-4 gene, exhibits complementarity to and consequently regulates the expression of the lin-14 protein (4). miRNAs are part of the small non-coding RNA family that are endogenous, single-stranded RNAs with an average length of 20–22 nucleotides, known to regulate gene expression in many physiological processes (5, 6). Studies have shown that miRNAs play a pleiotropic role acting as tumor suppressors and/or oncogenes in different cancers (7, 8). Numerous studies have now implicated miRNAs in every step of brain metastasis beginning from epithelial-mesenchymal transition to colonization in the brain parenchyma (9–29).

Brain organotropism in breast cancer is influenced by several factors: breast cancer subtype, molecular features of circulating tumor cells, extracellular vesicle-derived miRNA expression profile, tumor microenvironment, and the ability of breast cancer cells to penetrate the blood-brain barrier (BBB) (30). BBB is a specialized neurovascular unit adjoining blood capillaries with brain parenchyma, comprising of brain microvascular endothelial cells (BMECs), astrocytic end-feet, and pericytes (31). BMECs line the luminal and abluminal membranes and are held together by tight and adherens junctions (32). They tightly regulate the transport of cells and molecules from blood to the brain parenchyma. BMECs lack leukocyte adhesion molecules and have a higher concentration of mitochondria, which limits the influx of immune cells from blood into central nervous system (CNS) and indicates the prevalence of high-energy requiring role of BMECs respectively (33). In response to pathological changes in the CNS, astrocytes undergo molecular, functional, and morphological transformation and are termed as “reactive astrocytes”. Studies have shown the reactive astrocytes stimulate BMECs through secretion of *SERPINA3* by activating NF-κB/STAT3 signaling axis (34). Pericytes play a few roles in the maintenance of BBB integrity including regulating microvascular stability, angioarchitecture, and clearance of foreign proteins and tissue debris (35).

The interactions between tumor cells and brain microenvironmental cells, primarily astrocytes and microglia, facilitate various stages of metastasis. Reactive astrocytes through the secretion of inflammatory chemokines such as interferon-α (IFNα) and Ciliary Neurotrophic Factor promote tumor growth by activating transcriptional and cell survival pathways (36–38). Microglial cells, known as the resident macrophages of the CNS, are often polarized from M1 to M2 microglia to secrete immunosuppressive chemokines (39). These modulations in the environment play a key role in promoting the growth of brain metastasis. Through regulating the brain-metastatic microenvironmental cells and their interaction with breast cancer cells, miRNAs can influence breast cancer metastasis to the brain and progression within the brain.

## 2 MicroRNA biogenesis and mechanism of action

The biogenesis of miRNAs is classified into canonical and non-canonical pathways. The canonical pathway begins with the transcription of a hairpin-containing primary miRNA (pri-miRNA) in the nucleus (8). The pri-miRNA transcript is then cleaved by the microprocessor complex containing DiGeorge Syndrome Critical Region 8 (DGCR8) and Drosha to form the precursor miRNA (pre-miRNA). DGCR8 is an RNA-binding protein that recognizes and binds N6-methyladenosine GGAC motif in the pri-miRNA, and the RNase III enzyme, Drosha, recognizes and cleaves the base of the hairpin structure (40, 41). The pre-miRNA is transported from the nucleus to the cytoplasm through an exportin5/RanGTP complex where RNase III endonuclease Dicer cleaves the hairpin loop structure and leads to the formation of mature double-stranded miRNA (40, 42, 43).

There are two non-canonical pathways: Drosha/DGCR8-independent and Dicer-independent pathways. In the former pathway, (mirtrons) RNAs are exported to the cytoplasm through exportin 1 without undergoing Drosha processing; and in most cases, the 3p strand is loaded onto the AGO protein due to the presence of a 7-methylguanosine cap at the 5’ end (44). In the Dicer independent pathway, shRNA transcripts are processed by Drosha/DGCR8 complex and exported to the cytoplasm by exportin5/RanGTP where they are loaded onto AGO2 and processed (45, 46).

miRNAs binding to a specific seed sequence at either the 3’ or 5’ untranslated region of the target mRNA can lead to mRNA degradation or translational repression, leading to gene silencing (47–50). miRNAs can also bind at the promoter region of target mRNAs leading to transcriptional activation (51). miRNAs can regulate multiple biological pathways such as cell proliferation, cell death, immune evasion, invasion, metastasis, and angiogenesis. miRNAs are classified as tumor suppressors or oncogenes depending on their target gene and cell type (8).

## 3 Development of brain metastases

For the initiation of metastasis to occur, cancer cells undergo epithelial-mesenchymal transition (EMT) demonstrated by an increase in self-renewing stem cells, anoikis resistance, and dissemination (52–56). TWIST1, SNAIL1, and SLUG are some of the most heavily studied transcription factors in the context of breast cancer metastasis (57). Under regulation of pathways like the Notch signaling pathway, these transformed cells can penetrate the vascular endothelium where endothelial cells promote membrane remodeling and cancer cells enter blood vasculature (58–61). An important part of metastasis is the development of a tumor-supportive environment in distant target organs. Cancer cells prime a secondary site by secreting tumor-promoting extracellular vesicles and inflammatory chemokines, forming the premetastatic niche (62, 63). Extravasation into the brain requires tampering with the BBB permeability. The cross-talk between cancer cells and BMEC is stimulated by the expression of cellular adhesion molecules (E-selectin, VCAM-1) on cancer cells and degradation of the BBB by matrix metalloproteinases (64–66). Extravasation is followed by mesenchymal-epithelial transition (MET) or partial MET lending a higher aggressive phenotype to the cancer cell (67–69). Reactive astrocytes play a dichotomous role by initially inhibiting brain metastases and switching to a pro-metastatic role in later stages (37, 62, 70–72). Glial cells such as tumor-associated microglia/macrophages lend a supportive hand to cancer cells by stimulation of TGF-β1 signaling pathway (73).

## 4 miRNAs implicated in the cross-talk between breast cancer cells and brain cells

**Figure 1:**
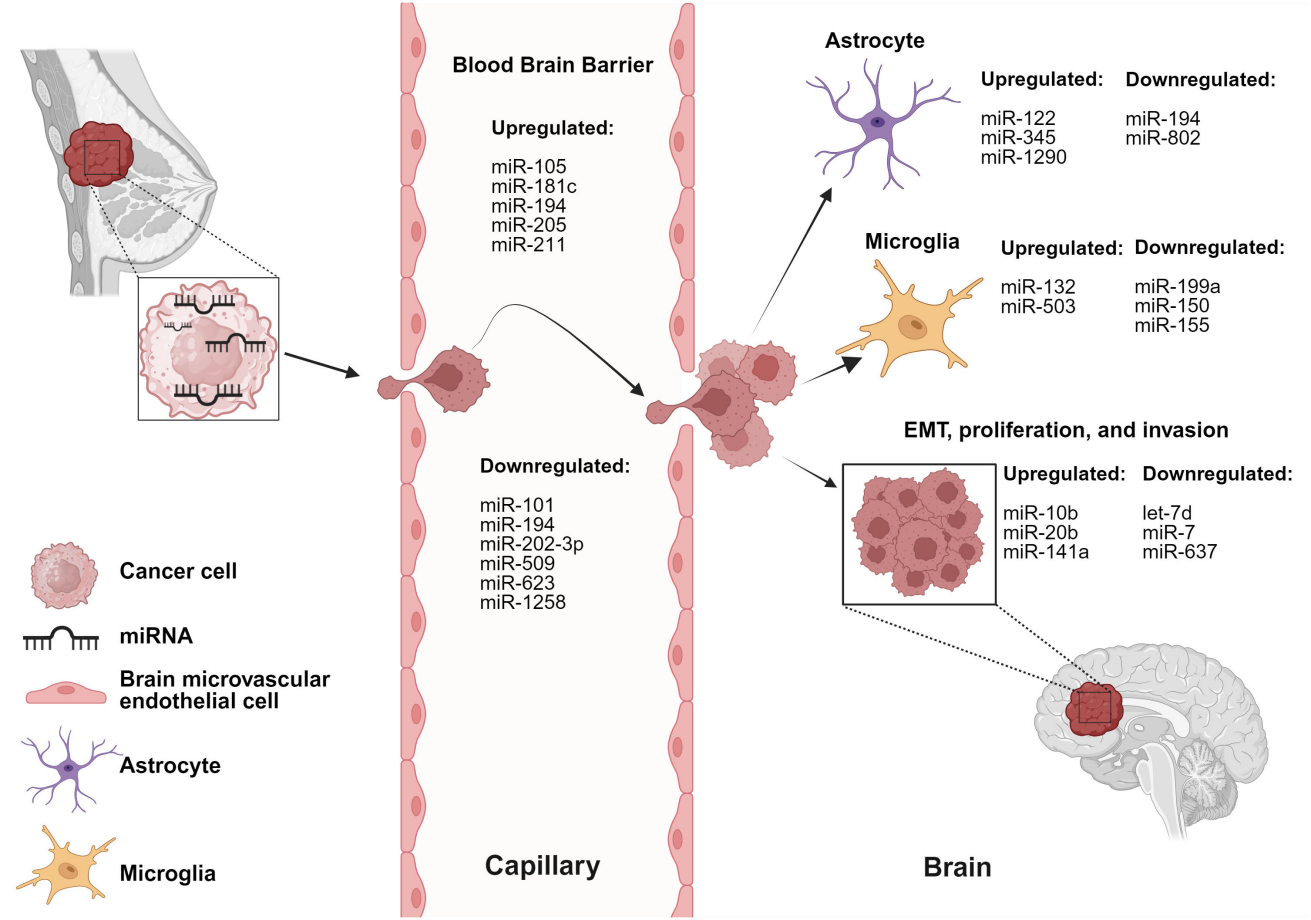
miRNAs are involved in dysregulation of various cells and stages of BCBM. BC-derived miRNAs alter functionality of brain and BBB microenvironmental cells. Created with BioRender.com.

The cross-talk between breast cancer cells and astrocytes/microglia at any stage of brain metastasis leads to microenvironmental modulation that subsequently facilitates the progression of brain metastasis. miRNAs involved in these interactions are listed in **Table 1** and depicted in **Figure 1**. 

**Table 1:** miRNAs mediate dysregulation of various cells and stages of BCBM.

miRNA	Activity	Validated Target(s)	*In-vitro* Models	Additional Model(s)	Reference
let-7d	Promote brain metastatic colonization	Pdgfb/PGDFA	4T1, D2A1, MDA-MB-231, MDA-MB-231-BrM2	Mouse	(74)
miR-7	Suppress brain metastases formation by inhibiting proliferation, invasion, and transmigration of CSCs	KLF4	MDA-MB-231, 231BoM-1833, 231BrM-2a, CN34, CN34-BoM2d, CN34-BrM2c, MCF7, MCF7-BoM2d	Mouse	(75)
miR-10b	Promote invasion of BC cells	No specific target	MDA-MB-231, MDA-MB-468	Patient samples	(76)
miR-20b	Promote colony formation and invasion of BC cells	No specific target	MCF-7, MDA-MB-231, MDA-MB-231-brain and bone derivatives	Patient samples	(77)
miR-101-3p	Promote extravasation and trans-endothelial migration of BC cells	COX2	MCF-7, MDA-MB-231, MDA-MB-231-BrM2, MDA-MB-231-TGL	N/A	(78)
miR-105	Promote tumor cell invasion and disrupt vascular endothelium	ZO-1	MDA-MB-231, MCF-10A, MCFDCIS, MDA-231-HM, Primary HMVECs	Patient samples	(79)
miR-122	Inhibit glucose uptake by astrocytes	PKM	MCF10A, MDA-MB-231	Mouse	(80)
miR-132-3p, miR-199a, miR-150 and miR-155	Promote tumor angiogenesis and colonization of BC cells	MET		Patient samples	(81)
miR-181c	Downregulate PDPK1 promoting BBB destruction	PDPK1	MDA-MB-231-luc-D3H1, MDA-MB-231-luc-D3H2LN, BMD2a, BMD2b	Mouse	(82)
miR-194 and miR-802	Upregulate MEF2C highly expressed in peritumoral astrocytes promoting cross-talk	MEF2C	4T1	Mouse, patient samples	(83)
miR-194, miR-181a-1-3p, miR-205,	Mediate cross-talk between BC cells and BMECs	No specific target	b.End5, 4T1	Mouse	(84)
miR-200 family (miR-141)	Induce MET and brain metastatic colonization	E-cadherin	SUM149, SUM159, MDA-IBC3, MDA-231, MCF7	Mouse, patient samples	(18)
miR-202-3p	Induce MMP-1 and promote extravasation of BC cells	MMP-1	MCF-7, MDA-MB-231-TGL, MDA-MB-231-BrM2, hCMEC/D3	Mouse	(85)
miR-211	Promote early colonization and BBB adherence through regulation of SOX11/NGN2 axis	SOX11/NGN2	MDA-MB-231, HCC1806, LM2-4175, BrM-831	Mouse	(86)
miR-345	Induce cancer progression through cross-talk and niche formation	KISS1, E-cadherin	CN34TGL, MDA-MB-231, CN34Br, MDA231Br	Mouse	(87)
miR-503	Induce M1 to M2 polarization of microglia	STAT3/NF-κB pathways	MCF7, ZR75-1, SKBR3, MDA-MB-231, SKBrM3, MDA-MB-231BrM2a, SIM-A9	Mouse, patient samples	(26)
miR-509	Suppress RhoC and TNFα mediated transmigration and invasion of BC cells	RhoC, TNF-α	MDA-MB-231, MDA-MB231BrM-2a, CN34, CN34-BrM2c, MCF7, MDA-MB-231-HM, 293TN, mBrEC	Mouse, patient samples	(88)
miR-623	Promote extravasation and trans-endothelial migration of BC cells	MMP-1	MDA-MB-231-BrM2, MDA-MB-231-TGL, hCMEC/D3	N/A	(89)
miR-637	Promote autophagy and invasion of TNBC cells	STAT3	MCF10A, BT549, MDA-MB-361, MDA-MB-453, MDA-MB-, MDA-MB-468, SUM-159, HCC-1806	Mouse, patient samples	(90)
miR-1258	Inhibit Heparanase to promote invasion	HPSE	MDA-MB-231BR1, MDA-MB-231BR3, SUM-225, SUM-149, HMEC, MCF-10A	Mouse, patient samples	(25)
miR-1290	Activate astrocytes in the brain metastatic microenvironment via the FOXA2→CNTF axis	FOXA2	MDA-MB-231, MDA-231-BRM, SKBR3, CN34, SKBRM-tGLI1, immortalized human astrocytes	Mouse	(36)

miR-122 is upregulated in the conditioned media of breast cancer cells (80). Uptake of breast cancer-derived miR-122 by astrocytes led to a reprogramming of glucose uptake, notably through the downregulation of PKM1/2 and GLUT1, resulting in decreased uptake of 2-NBDG (a fluorescent glucose analogue). The authors reported reduced glucose uptake by tumor cells led to inhibition of tumor cell proliferation in primary tumors, while simultaneously supporting metastatic tumor cell colonization in the pre-metastatic niche (80).

miR-194 and miR-802 were downregulated in plasma samples collected from brain metastasis model of a 4T1-injected mice (83). *MEF2C* was validated as a target gene using an *in vivo* model through immunofluorescence staining. *MEF2C* was found to be highly expressed in established metastatic cells in the brain parenchyma and peritumoral astrocytes. These findings were further validated in human TNBC brain metastases samples (83). Moreover, miR-802 inhibits FoxM1 decreasing the proliferation of breast cancer cells and miR-194 inhibits proliferation and migration of breast cancer cells, supporting the tumor-suppressive roles of these miRNAs (91, 92).

Breast cancer-derived miR-345 expression is upregulated due to increased astrocytic secretion of CCL2 and CXCL12 (87). miR-345 downregulates KISS1 which in turn leads to localization of breast cancer cells in the brain microenvironment (87). Another study reports that miR-345-mediated KISS1 transcriptional inhibition plays a role in promoting autophagy and invasiveness of breast cancer cells (87, 93).

miR-1290 is upregulated in the sera of breast cancer patients (36). miR-1290 activates astrocytes and promotes cancer stemness factors, subsequently aiding the progression of BCBM. miR-1290 binds to FOXA2 and induces transcriptional repression, leading to upregulation of CNTF expression in astrocytes. Astrocytes activated by miR-1290 promoted intracranial growth of co-implanted breast cancer cells in the brain *in vivo* (36).

miR-199a, miR-150, and miR-155 are downregulated, whereas miR-132-3p is upregulated in tissue obtained from BCBM patients (81). The authors report an increase in miR-132-3p correlated with improved brain metastasis-free survival (BMFS) and overall survival (OS) whereas miR-199a, miR-150 and miR-155 correlated with poorer BMFS and OS. *MET* was identified as a target oncogene and reported to be overexpressed in microglial cells responsible for tumor angiogenesis and colonization of breast cancer cells (81). miR-503 is upregulated in the sera of BCBM patients and is reported to promote the M1 to M2 polarization of microglia, demonstrated by increased phosphorylation of STAT3 along with decreased phosphorylation of NF-κB (26). Taken together, these findings suggest an important role for miRNAs in regulating the brain microenvironmental cells.

## 5 miRNAs implicated in the cross-talk between breast cancer cells and BBB cells

A pivotal aspect of brain metastasis involves the disruption of the BBB integrity. Thus, studying the interactions between breast cancer cells and BBB cells is of utmost importance. miRNAs involved in mediating cross-talk between breast cancer cells and BBB cells are listed in **Table 1**.

miR-101-3p is decreased in BCBM leading to increased expression of COX-2 and stimulation of COX-2/MMP-1 signaling pathway, promoting trans-endothelial migration of breast cancer cells and extravasation across the BBB (78). miR-105 downregulates ZO-1 expression in endothelial cells hindering the integrity of endothelial and epithelial tight junctions leading to trans-endothelial invasion of breast cancer cells. Disruption of the endothelial barriers and increased vascular permeability promoted distant metastases formation in lung and brain (79). miR-181c is increased in sera of brain metastasis patients compared to non-brain metastasis patients. The study reports that miR-181c regulates the expression of PDPK1 in BMECs, where PDPK1 plays a role in localization of N-cadherin and actin filaments in BMECs. Delocalization of actin in the BMECs plays a role in BBB dysregulation (82).

miR-202-3p is decreased in brain-tropic breast cancer cell lines. The authors report restoration of miR-202-3p leading to MMP-1 suppression results in inhibited extravasation of BCBM cells (85). MMP-1 has previously been reported to promote trans-endothelial migration of breast cancer cells by degrading endothelial junctions in BMECs and permeabilizing the endothelial barrier. One study reported the upregulation of miR-205 and miR-181a-1-3p along with downregulation of miR-194 in co-culture models. The authors report overexpression of miR-181a-1-3p results from the interaction between breast cancer cells and BMECs, and that BMECs contribute to the downregulation of miR-194, while breast cancer cells upregulate the expression of miR-205 (84).

miR-211 is upregulated in the brain-tropic TNBC cells and human breast cancer tissue from TNBC and non-TNBC patients. miR-211 overexpressing breast cancer cells promote brain metastases *in vivo*; inhibition of miR-211 with anti-miR-211 treatment suppresses brain metastases *in vivo*. Increased expression of miR-211 promoted migration and invasion of TNBC cells and enhanced adherence of cancer cells to the BBB through downregulation of SOX11/NGN2 axis (86).

miR-509 is downregulated in brain metastases compared to primary breast tumors and targets RhoC, a critical mediator of metastasis and invasion. Additionally, miR-509 suppressed the trans-endothelial migration of breast cancer cells and contributed to the suppression of MMP9 via modulation of RhoC (94). miR-509 also indirectly represses TNFα leading to decreased BBB permeability (88). miR-623 is downregulated in brain metastatic lesions in comparison to primary breast tumors. MMP-1 is known to play a significant role in promoting extravasation of TNBC cells into the brain endothelium and is suppressed by miR-623. This study reports the restoration of miR-623 inhibits trans-endothelial migration of brain-tropic TNBC cells, thereby suppressing BCBM (89). Downregulation of miR-1258 was associated with an increase in HPSE levels in BCBM cell lines, paired primary breast tissue, and BCBM tissue. miR-1258 expression leads to a decrease in HPSE levels and HPSE-related proteins: p-Akt, p-EGFR, MMP-9, COX2 consequently, leading to inhibition of brain metastasis by limiting breast cancer cell invasion (25). These studies are further proof of the crucial role of miRNAs in maintenance of BBB integrity.

## 6 miRNAs implicated in EMT, invasion, and colonization of breast cancer cells in BCBM

EMT, invasion, and colonization serve an essential role in metastasis of cancer cells. miRNAs involved in these processes are briefly described in **Table 1**.

miR-7 is downregulated in mammospheres and brain-tropic breast cancer cell lines compared to parental cells (75). *KLF4* is a miR-7 target gene and *in vivo* studies report miR-7 inhibits the expression of *KLF4* downregulating the proliferation, invasion, and transmigration of brain-tropic cancer stem cells (CSCs). The miR-7 and *KLF4* correlation was further validated in human samples from primary breast tumor and brain metastatic lesions, and the authors suggest interaction between CSCs and brain cells promotes formation of a pre-metastatic niche (75).

miR-10b is significantly upregulated in tumor samples of BCBM patients when compared to primary breast tumors without brain metastasis (76). It was reported that higher levels of miR-10b were correlated with increased invasiveness of breast cancer cells (76). miR-20b is increased in brain metastatic lesions of BCBM patients compared to breast cancer patients without brain metastasis (77). miR-20b is also upregulated in brain-tropic breast cancer cells compared to bone-tropic breast cancer cells highlighting brain-tropism of miR-20b. miR-20b overexpression resulted in increased colony formation and invasiveness of breast cancer cells (77).

let-7d is downregulated in brain metastatic breast cancer cells and regulates PGDFA expression (74). PGDFA inhibition leads to decreased brain metastases formation in mice models due to the loss of autocrine proliferation loop activity which promotes metastatic colonization. The authors also reported HIF1 activity is negatively regulated by let-7d (74). miR-141 expression is upregulated in sera from BCBM patients (18). Upregulation of miR-141 was correlated with increased E-cadherin expression, which suggests miR-141 plays a role in EMT. The role of miR-200 family (miR-200a, miR-200b, miR-200c, miR-141, miR-429) in EMT has been well established in various metastatic models and the authors propose a possible role in breast cancer brain metastatic colonization (18). Moreover, circKIF4A sponges miR-637 to suppress its expression in brain metastatic lesions compared to primary breast tumors. miR-637 inhibits STAT3, therefore, miR-637 inhibition increases STAT3 protein levels and promotes the brain metastatic properties of TNBC cells through autophagic activation (90). These studies suggest that miRNAs play key roles in regulating multiple stages of BCBM; however, further investigations into these mechanisms are necessary.

## 7 Application of miRNAs in cancer therapeutics

The dismal overall survival of patients with BCBM is partly due to the lack of early biomarkers and targeted BBB-penetrant therapies (95–97), and miRNAs could possibly be used to address both deficiencies. The ubiquitous presence of miRNAs in peripheral blood, urine, and saliva makes it a highly valuable and non-invasive biomarker of disease burden, progression, treatment response, and resistance (98–100). Currently, there are several clinical trials assessing the potential application of miRNAs as biomarkers. Project CADENCE (NCT05633342) is a cohort study aimed at investigating miRNA expression along with other biomarkers and ultimately developing *in-vitro* diagnostic assays for screening nine highly prevalent cancers: breast, colorectal, lung, prostate, liver, pancreatic, gastric, ovarian and esophageal (101). Oncoliq US (NCT06439940) is actively recruiting for a prospective cohort study to identify early diagnosis markers for breast cancer utilizing liquid biopsies and miRNAs (102). Another prospective cohort study (NCT05417048) at Peking University is currently investigating the performance of a blood-based assay utilizing miRNAs to differentiate between benign and malignant breast disease (103). MiraKind is currently running a prospective cohort study (NCT02253251) to validate the role of mutations at miRNA binding sites in breast and ovarian cancer patients (104). A randomized diagnostic clinical trial (NCT04516330) is investigating the role of an 84-miRNA panel in predicting multicentricity in breast cancer (105). City of Hope Medical Center conducted a cohort study (NCT01231386) to perform miRNA profiling in patients undergoing treatment for locally advanced or inflammatory breast cancer (106).

miRNA profiling has also been used as a non-surgical tool to differentiate between medulloblastoma, glioblastoma, BCBM, and lung cancer brain metastasis (107). High expression of miR-200 family in the cerebrospinal fluid of brain metastasis patients helps differentiate between cases of brain metastasis and glioblastoma (108).

Therapeutic miRNA development is being explored given the significant roles that miRNAs play in dysregulation of multiple genes leading to tumor initiation, progression, and metastasis (95–97). MRG-106, an inhibitor of miR-155, was investigated for the treatment of cutaneous T-cell lymphoma, mycosis fungoides subtype. The Phase I study reported tolerability and reduction in the Composite Assessment of Index Lesion Severity score and modified Severity Weighted Assessment Tool used to measure skin lesions/disease (109). However, the study by Miragen Therapeutics was discontinued during Phase II (NCT03713320) due to business reasons. Miragen Therapeutics also completed a Phase I study (NCT03603431) with MRG-110, a miR-92a inhibitor, which was investigated in healthy volunteers and was reported to augment wound healing and angiogenesis (110). TransCode Therapeutics recently entered a Phase I/II dose-escalation study (NCT06260774) with TTX-MC138, a miR-10b inhibitor, which has previously been implicated in metastatic lesions arising from advanced solid tumors (111, 112). MRX34, miR-34a mimic, was investigated in patients with unresectable primary liver cancer, hematological malignancies and advanced solid tumors (NCT01829971). MiRNA therapeutics reported treatment with MRX34 demonstrated some clinical activity, however, treatment-associated severe adverse events led to termination of the study (113). CDR132L, a selective miR-132-3p inhibitor, is currently in Phase II clinical trial (NCT05350969) for patients with reduced Left Ventricular Ejection fraction post-myocardial infarction (114). In 2019, Regulus Therapeutics announced pre-clinical success of RGLS5579, an anti-miR-10b, in combination with temozolomide (TMZ) in glioblastoma animal models (115). Regulus Therapeutics reported the median survival rate of glioblastoma-bearing mice models treated with anti-miR-10b, anti-miR-10b in combination with TMZ, and TMZ alone increased by 18%, >120%, and 27% respectively. Combination of tumor suppressive miRNAs with conventional chemotherapy is another promising avenue. Tumor suppressive miR-770 inhibited doxorubicin resistance in TNBC and promoted sensitivity to trastuzumab in HER2-positive breast cancer (116, 117). Overexpression of miR-298 sensitizes doxorubicin-resistant breast cancer cells to treatment by targeting MDR1 (118). Other studies have also reported targeting of ABCG2 overexpression of miR-181a or miR-328 in breast cancer cells led to increased sensitivity to mitoxantrone (119, 120). miRNAs may be the key to improving targeted therapeutics for cancer patients; however, it is worth noting that many Phase I and II clinical trials have been halted in the past due to severe adverse effects (96, 121, 122). Nonetheless, these studies highlight the importance of further investigations into the role of miRNAs as biomarkers and for the advancement of miRNA incorporation into cancer therapeutics.

## 8 Discussion

There has been a rise in incidence of BCBM, due to significantly advanced and effective therapies that prolong patient survival. This extended survival period allows latent metastatic cells greater opportunity to penetrate the BBB and colonize in the brain parenchyma. Given the rise in frequency and limited treatment opportunities of brain metastasis, there is an urgent need for new predictive, diagnostic, and prognostic biomarkers to assess brain metastasis. miRNAs are small but mighty in the regulation of every step of brain metastasis starting from the cancer stemness genes, genes responsible for intravasation and extravasation into a foreign site, organ tropism, and colonization-related genes. The specificity of dysregulated miRNAs in brain metastasis from various primary tumors can be utilized to differentiate tumor types and identify the origin of primary tumors in unknown cases. Certainly, further research aimed at identifying novel miRNAs, elucidating their biological functions, and uncovering their target genes will significantly enhance our understanding of the role miRNAs play in metastases formation and progression. This will, in turn, lay the groundwork for the advancement of miRNA-related approaches for cancer prognosis, diagnosis, and treatment.

## Author contributions

MK: Writing – original draft, Conceptualization. GW: Writing – review & editing. CZ: Writing – review & editing. MN: Writing – review & editing, Visualization. H-WL: Writing – review & editing, Supervision, Resources, Funding acquisition, Conceptualization.
